# KRT in Brazil: A Retrospective Cohort Study Based on Analysis of the Brazilian Public Health System

**DOI:** 10.34067/KID.0000000000000539

**Published:** 2024-08-06

**Authors:** Guilherme Palhares Aversa Santos, Ricardo Sesso, Jocemir Ronaldo Lugon, Precil Diego Miranda de Menezes Neves, Abner Mácola Pacheco Barbosa, Naila Camila da Rocha, Luis Gustavo Modelli de Andrade

**Affiliations:** 1 Department of Internal Medicine–UNESP, Univ Estadual Paulista, Botucatu, Brazil; 2 Division of Nephrology, Department of Medicine, Universidade Federal de São Paulo, São Paulo, Brazil; 3 Division of Nephrology, Department of Medicine, Universidade Federal Fluminense, Rio de Janeiro, Brazil; 4 Nephrology Division, University of São Paulo–USP, São Paulo, Brazil; 5 Nephrology and Dialysis Center-Hospital Alemão Oswaldo Cruz–São Paulo, São Paulo, Brazil

**Keywords:** chronic dialysis, chronic hemodialysis, CKD, chronic kidney failure, chronic renal insufficiency, clinical epidemiology, peritoneal dialysis

## Abstract

**Key Points:**

- A large comprehensive analysis of patients undergoing KRT within Brazil's Public Health System from 2015 to 2023.
- We reported an increase in the age at which dialysis began and a decline in the adoption of peritoneal dialysis over the years.
- We showed better hemodialysis adequacy as measured by single-pool Kt/V.

**Background:**

Brazil has the largest public health system providing universal coverage for chronic dialysis. The objective was to describe the number, sociodemographic, and clinical characteristics of patients undergoing KRT by dialysis within the public health system in Brazil.

**Methods:**

We carried out a retrospective cohort study analyzing the database from the Brazilian Public Health System, focusing on procedures related to KRT. The study encompassed both prevalent and incident patients who underwent KRT in Brazil between 2015 and 2023.

**Results:**

We observed an increase in the number and prevalence rate of dialysis patients from 2015 to 2023. We also noticed an increase in the age at dialysis initiation and in the prevalence of mixed-race patients and a reduction in the proportion of those undergoing peritoneal dialysis and with arteriovenous fistula. We identified an upward trajectory in the values of single-pool Kt/V over the years, contrasting with a decline in hemoglobin levels. The overall estimated prevalence rate of dialysis patients increased from 654 per million population (pmp) to 792 pmp over the years. The survival rates of incident patients undergoing KRT at 12 and 96 months were 81% and 60%, respectively.

**Conclusions:**

We reported an increase in the age at which dialysis began and a decline in the adoption of peritoneal dialysis over the years. Although there have been some improvements over the years resulting in better adequacy of hemodialysis as measured by Kt/V, controlling certain parameters, such as hemoglobin levels, has remained challenging.

## Visual Abstract

**Figure:**
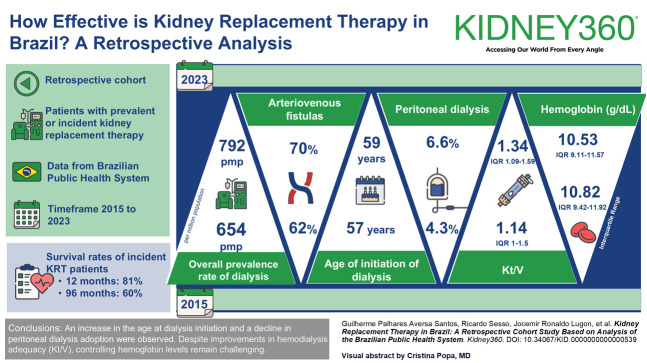

## Introduction

Brazil is a high middle-income country with a large public health system (Brazilian Unified Health System [SUS]) implemented in 1990 that provides universal coverage for chronic dialysis and primary and specialized medical care, allowing free access to many high-cost medications for the population.^1^ Over the past decades, the SUS has been growing despite the ongoing struggle with underfunding. Approximately 80% of KRT procedures in the country are reimbursed by the public health system.^2^ Brazil has been ranked third in the world in terms of the number of patients undergoing dialysis, reaching 153,831 patients on maintenance dialysis in 2022, of a population of 203,080 million inhabitants in the same year.^3^

The Brazilian Society of Nephrology provides epidemiological data on patients undergoing KRT in Brazil through an annual survey.^3^ However, because this is a voluntary initiative, the response rate has varied between 28% and 38%, figures that call for some caution regarding the interpretation and generalization of data.^4^

Department of Informatics of the Unified Health System (DATASUS) comprehensive Brazilian Public Health System dataset yields a wealth of information which is mandatory for reimbursement and essential for epidemiological purposes.^5^ Despite this, there have been few detailed studies describing the clinical and epidemiological aspects of patients undergoing KRT in Brazil supported by the public health system.^6,7^ The objective of this study was to describe the number, sociodemographic and clinical characteristics, and laboratory parameters of patients undergoing KRT by dialysis in the Brazilian Public Health System from 2015 to 2023. Crude survival rates for incident patients were also reported.

## Methods

### Population

We carried out a retrospective cohort study using the database from the Brazilian Public Health System related to KRT procedures by dialysis using claims data. We included all prevalent and incident patients who underwent hemodialysis and peritoneal dialysis procedures in Brazil between 2015 and 2023. Ethical approval was waived because of the anonymized and publicly available nature of the database.

### Inclusion and Exclusion Criteria

All prevalent and incident patients who underwent dialysis-related KRT procedures in Brazil between January 2015 and December 2023 were included.

### Data Retrieved

The data were provided by DATASUS,^5^ a health information system maintained by the Brazilian Ministry of Health. DATASUS provides information about the SUS. Data on dialysis procedures are available through the outpatient procedures system (Ambulatory Procedures System)^8^ and are linked to reimbursement to the facilities providing dialysis therapy for outpatients with chronic renal failure. The information must be filled out by dialysis facilities every month. In the original system, records are arranged by individual procedures, resulting in multiple records for the same patient. To circumvent the problem, we developed a computerized algorithm to retrieve data for individual patients. By using a unique identification code provided by the Brazilian Public Health System, we were able to identify each case and rearrange the procedures per patient. Our mathematical/electronic system prevented duplicated registries, and by consolidating claims per patient, inclusion in the final database for analysis was accomplished without that risk. Patients with AKI are not registered in the Ambulatory Procedures System and, therefore, were not included in this study.

### Clinical Variables

The variables studied included age at which the dialysis began, sex, ethnicity, Brazilian region, type of KRT (hemodialysis and peritoneal dialysis), type of the last vascular access used (arteriovenous fistula, short-term hemodialysis catheter, and long-term tunneled hemodialysis catheter), and serology for HIV, hepatitis B, and hepatitis C. We also retrieved blood hemoglobin, serum phosphorus, parathormone (PTH), single-pool Kt/V, and serum albumin. The biochemical examinations were analyzed using the mean of all available values for each patient during follow-up. In general, the biochemical examinations, except for PTH and serum albumin, were collected monthly and recorded in the same reimbursement form, as required by Brazilian legislation. PTH and serum albumin were tested every 3 months.

The self-declared ethnicity was classified as reported by the patient as White, Brown/Black (mixed race), Yellow (Asian), and Indigenous. The Brazilian regions were classified as North, Northeast, Midwest, Southeast, and South.

To assess the number of KRT centers, we used the Brazilian National Register of Legal Entities, a unique code assigned to each center. To determine the number of Brazilian cities offering KRT, we relied on the Brazilian Institute of Geography and Statistics (IBGE) code.

The government reimburses the dialysis provider with a predetermined fee for the service, which is determined based on either the value per hemodialysis session or per peritoneal dialysis per patient-month. Reimbursements were retrieved by summing the values of all procedures (dialysis sessions) and represent the total government expenses for each procedure per patient/year in US dollars. We converted the local currency (Brazilian Real) to US dollars using the exchange rate from the first of July of each year.

### Adequacy Metrics

We classified the monthly examinations into reference ranges according to the Kidney Disease Outcomes Quality Initiative (KDOQI) guidelines.^9^ The adequacy of hemodialysis was assessed using the single-pool Kt/V (spKt/V). We did not evaluate Kt/V for patients on peritoneal dialysis. We considered the following values to classify ranges of adequacy according to the KDOQI: serum phosphorus between 3.5 and 5.5 mg/dl; PTH levels between 150 and 600 pg/ml; hemoglobin levels between 10 and 12 g/dl; and spKt/V higher than 1.2. Values within these ranges were considered in range, whereas those outside of the specified ranges were considered out of range.

### Brazilian Data

We used data from the IBGE^10^ as the source of the total Brazilian population. From 2015 to 2021, the figures were estimated on the basis of the 2010 census, while in 2022, the number was retrieved from the Brazilian demographic census carried out that year.^10^ In 2022, it was noticed that the population in the past years had been overestimated, leading to an underestimation of the prevalence rates of KRT between 2015 and 2021. We estimated the prevalence rate for patients on KRT reported as the number of patients per million population and calculated as the total number of dialysis patients during the year divided by the Brazilian population on July 1.

### Outcome

The outcomes analyzed were mortality, transfer to another center, and recovery of renal function. Those outcomes were related to 1 year in prevalent patients. In addition, we evaluated survival in incident patients who initiated dialysis from January 2015 onward closing the observation period on December 31, 2023.

### Statistical Analysis

Descriptive epidemiological data were performed using median and percentiles as the measures of central tendency. Categorical variables were presented in number and frequency. We used joinpoint regression analysis to estimate piecewise log-linear trends in rates over time. This method is particularly valuable for evaluating alterations in time series data, effectively pinpointing shifts in population structure and determining the timing of these changes.^11^ An annual percentage change with a 95% confidence interval (CI) was calculated for each identified trend segment. In addition, the average annual percentage change (AAPC) was computed to provide a single summary measure characterizing the overall rate of change across the entire study period. The annual prevalence rate of dialysis therapy was calculated as the number of patients on dialysis during the year, divided by the total Brazilian population on July 1. Crude mortality rate was calculated as the number of deaths during the year divided by the total number of patients undergoing dialysis in the same year. To analyze survival, we used the Kaplan–Meier method; the initial date was the date of beginning of dialysis therapy, and the final date was the date of death or the end of the study or the date of the last follow-up alive, whichever occurred first. Analyses were performed using R version 4.1.2.

## Results

**Figure 1:**
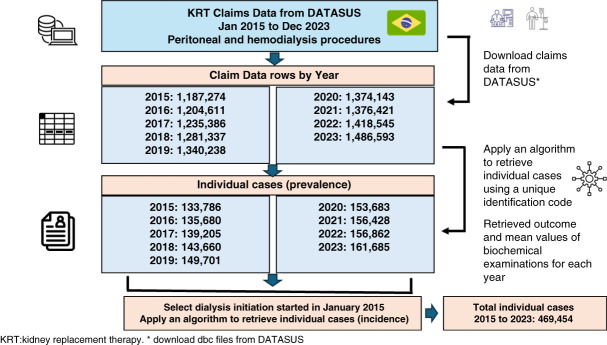
**Flow chart of patients on KRT through dialysis in the Brazilian Public Health System from 2015 to 2023.** The KRT procedures were extracted from claims data provided by DATASUS. Each row in the claims data represents a procedure, and we applied an algorithm to extract individual cases using a unique identification code. Biochemical values were retrieved as mean values. To identify incident cases from 2015 to 2023, we selected patients who initiated dialysis in January 2015 and applied an algorithm to retrieve individual cases. *Download dbc files from DATASUS. DATASUS, Department of Informatics of the Unified Health System.

For each year from 2015 to 2023, we collected the total number of claims data from DATASUS relating to peritoneal and hemodialysis procedures resulting in 11,904,548 rows of data. We merged the files by rows using the unique patient identification code resulting in 1,330,690 prevalent patients during the entire aforementioned period. This process resulted in the identification of the number of individual cases that performed KRT. In 2023 alone, we retrieved 1,486,593 rows of claims data resulting in the identification of 161,685 prevalent patients. In 2015, 133,786 patients received maintenance dialysis. For incident patients, we merged all data from 2015 to 2023 resulting in a dataset of 469,454 patients who initiated dialysis from January 1, 2015, onward (Figure 1).

**Figure 2:**
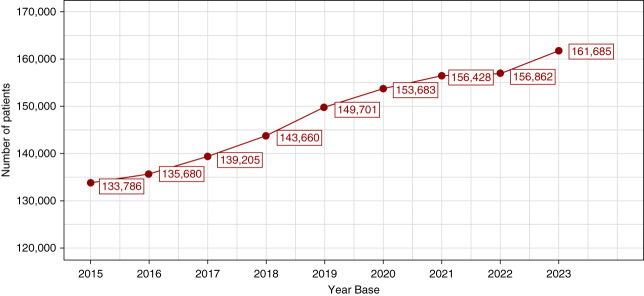
Number of patients on KRT by dialysis in the Brazilian Public Health System over the years (2015–2023).

There was a progressive increase in the number of patients undergoing KRT in Brazil from 2015 to 2023, except for 2022, during the coronavirus disease 2019 (COVID-19) pandemic, when it remained stable (Figure 2). The age at which the dialysis began also increased over the years, from a median (interquartile range [IQR]) of 57 (45–67) years in 2015 to 59 (47–68) years in 2023, AAPC 0.31% (95% CI, 0.28% to 0.36%), Tables 1 and 2. The percentage of patients older than 60 years increased from 43% to 48% during the same period, AAPC 3.57% (95% CI, 2.93% to 4.26%). The percentage of pediatric patients (younger than 18 years) decreased from 1.3% to 0.9%, AAPC −2.22% (95% CI, −2.67% to −1.66%). There was an increase in the percentage of mixed race patients over the years, ranging from 46% to 59%, AAPC 5.81% (95% CI, 4.92% to 6.39%) (Tables 1 and 2).

**Table 1:** Clinical characteristics and outcomes of patients undergoing KRT in the Brazilian Public Health System over the years ranging from 2015 to 2023

Characteristic	2015, *n*=133,786	2016, *n*=135,680	2017, *n*=139,205	2018, *n*=143,660	2019, *n*=149,701	2020, *n*=153,683	2021, *n*=156,428	2022, *n*=156,862	2023, *n*=161,685
**Age, yr**	57 (45–67)	57 (45–67)	57 (46–67)	58 (46–68)	58 (46–68)	58 (46–68)	58 (46–68)	58 (46–68)	59 (47–68)
Younger than 18	1689 (1.3%)	1581 (1.2%)	1581 (1.1%)	1500 (1.0%)	1478 (1.0%)	1429 (0.9%)	1430 (0.9%)	1422 (0.9%)	1380 (0.9%)
Older than 60	58,195 (43%)	59,718 (44%)	62,192 (45%)	65,278 (45%)	68,923 (46%)	71,737 (47%)	72,973 (47%)	73,524 (47%)	77,002 (48%)
**Sex**									
Female	56,000 (42%)	56,638 (42%)	57,918 (42%)	59,754 (42%)	62,170 (42%)	63,517 (41%)	64,920 (42%)	64,847 (41%)	67,017 (41%)
Male	77,786 (58%)	79,042 (58%)	81,287 (58%)	83,906 (58%)	87,531 (58%)	90,166 (59%)	91,508 (58%)	92,015 (59%)	94,668 (59%)
**Ethnicity**									
Mixed race	61,445 (46%)	62,641 (46%)	65,231 (47%)	69,479 (48%)	74,367 (50%)	77,379 (50%)	79,299 (51%)	86,787 (55%)	95,352 (59%)
Not informed	15,829 (12%)	16,230 (12%)	16,577 (12%)	15,768 (11%)	15,227 (10%)	15,013 (9.8%)	14,771 (9.4%)	6045 (3.9%)	259 (0.2%)
White	54,959 (41%)	55,189 (41%)	55,493 (40%)	56,566 (39%)	58,246 (39%)	59,184 (39%)	59,871 (38%)	60,678 (39%)	62,556 (39%)
Yellow/Indigenous	1553 (1.2%)	1620 (1.2%)	1904 (1.4%)	1847 (1.3%)	1861 (1.2%)	2107 (1.4%)	2487 (1.6%)	3352 (2.1%)	3518 (2.2%)
**Category**									
Hemodialysis	124,967 (93%)	127,372 (94%)	131,328 (94%)	135,959 (95%)	142,060 (95%)	146,172 (95%)	149,372 (95%)	150,045 (96%)	154,788 (96%)
Peritoneal dialysis	8819 (6.6%)	8308 (6.1%)	7877 (5.7%)	7701 (5.4%)	7641 (5.1%)	7511 (4.9%)	7056 (4.5%)	6817 (4.3%)	6897 (4.3%)
**Vascular access for hemodialysis**									
Long-term catheter	27,882 (22%)	29,025 (23%)	29,739 (23%)	30,704 (23%)	31,752 (22%)	32,176 (22%)	37,901 (25%)	37,706 (25%)	38,479 (25%)
Short-term catheter	9465 (7.6%)	9378 (7.4%)	10,591 (8.1%)	11,844 (8.7%)	14,070 (9.9%)	17,280 (12%)	18,030 (12%)	19,343 (13%)	20,777 (13%)
Arteriovenous fistula	87,620 (70%)	88,969 (70%)	90,998 (69%)	93,411 (69%)	96,238 (68%)	96,716 (66%)	93,441 (63%)	92,996 (62%)	95,532 (62%)
**Brazilian region**									
Midwest	10,871 (8.1%)	11,362 (8.4%)	11,393 (8.2%)	12,214 (8.5%)	12,342 (8.2%)	12,505 (8.1%)	13,065 (8.4%)	12,588 (8.0%)	13,055 (8.1%)
Northeast	33,444 (25%)	33,990 (25%)	35,974 (26%)	36,537 (25%)	39,212 (26%)	40,594 (26%)	41,162 (26%)	42,195 (27%)	44,303 (27%)
North	6901 (5.2%)	6664 (4.9%)	7233 (5.2%)	7709 (5.4%)	8401 (5.6%)	8724 (5.7%)	8861 (5.7%)	9548 (6.1%)	9606 (5.9%)
Southeast	62,771 (47%)	63,847 (47%)	64,393 (46%)	66,400 (46%)	67,912 (45%)	69,487 (45%)	70,189 (45%)	69,733 (44%)	71,748 (44%)
South	19,799 (15%)	19,817 (15%)	20,212 (15%)	20,800 (14%)	21,834 (15%)	22,373 (15%)	23,151 (15%)	22,798 (15%)	22,973 (14%)
Positive serology for HIV	1270 (0.9%)	1358 (1.0%)	1331 (1.0%)	1565 (1.1%)	1729 (1.2%)	1820 (1.2%)	2069 (1.3%)	2499 (1.6%)	1909 (1.2%)
Positive serology for hepatitis B	1214 (0.9%)	1078 (0.8%)	1067 (0.8%)	1194 (0.8%)	1320 (0.9%)	1179 (0.8%)	1216 (0.8%)	1743 (1.1%)	1110 (0.7%)
Positive serology for hepatitis C	4563 (3.4%)	4412 (3.3%)	4155 (3.0%)	4205 (2.9%)	4126 (2.8%)	3891 (2.5%)	3874 (2.5%)	4001 (2.6%)	3144 (1.9%)
Recovery of renal function^a^	940 (0.7%)	898 (0.7%)	1063 (0.8%)	1156 (0.8%)	1258 (0.8%)	1170 (0.8%)	1099 (0.7%)	1421 (0.9%)	1557 (1.0%)
Patients transferred between centers	2519 (1.9%)	2476 (1.8%)	2416 (1.7%)	2578 (1.8%)	2724 (1.8%)	2401 (1.6%)	2710 (1.7%)	3370 (2.1%)	3523 (2.2%)
1-yr mortality	15,371 (11%)	15,813 (12%)	15,623 (11%)	15,733 (11%)	16,166 (11%)	19,610 (13%)	21,405 (14%)	17,726 (11%)	16,661 (10%)
Total expenditure US dollars per patient per year	6793 (2,681–7401)	7731 (3,134–8390)	8604 (3,521–9374)	7421 (3,034–8042)	7207 (2,937–7784)	5649 (2,277–6065)	5058 (1,854–5523)	6216 (2,614–6727)	6906 (3,125–7403)

The categorical variables were described in number and percentage. The numeric variables were reported in median and percentiles (25% and 75%).

a Improvement in the GFR rendering dialysis therapy unnecessary.

**Table 2:** Annual percentage changes in clinical characteristics and outcomes of patients undergoing KRT patients in the Brazilian Public Health System over the years ranging from 2015 to 2023

Characteristic	Segment 1^a^	APC (95% CI)	Segment 2^a^	APC (95% CI)	AAPC (95% CI–Full Range)
**Age, yr**	2015–2020	0.38^b^ (0.34 to 0.54)	2020–2023	0.19^b^ (0.01 to 0.29)	0.31^b^ (0.28 to 0.36)
Younger than 18	2015–2018	−4.25^b^ (−6.66 to −2.62)	2018–2023	−0.97 (−1.18 to 1.28)	−2.22^b^ (−2.67 to −1.66)
Older than 60	2015–2020	4.38^b^ (3.65 to 7.18)	2020–2023	2.23 (−0.83 to 3.53)	3.57^b^ (2.93 to 4.26)
**Sex**					
Female	2015–2023	2.38^b^ (1.88 to 2.87)			2.38^b^ (1.88 to 2.87
Male	2015–2020	3.11^b^ (2.15 to 5.55)	2020–2023	1.63 (−0.9 to 2.98)	2.55^b^ (2.02 to 3.13)
**Ethnicity**					
Mixed race	2015–2021	4.79^b^ (1.83 to 5.96)	2021–2023	8.93^b^ (5.18 to 11.56)	5.81^b^ (4.92 to 6.39)
Not informed	2015–2021	2.84 (−10.37 to 24.96)	2021–2023	−85.27^b^ (−90.3 to −72.6)	−36.7^b^ (−42.3 to −28.1)
White	2015–2017	0.66 (−0.01 to 1.65)	2017–2023	1.91^b^ (1.64 to 2.65)	1.59^b^ (1.43 to 1.83)
Yellow/indigenous	2015–2020	5.88 (−0.38 to 8.92)	2020–2023	21.1^b^ (13.99 to 32.88)	11.35^b^ (9.15 to 13.38)
**Category**					
Hemodialysis	2015–2020	3.33^b^ (2.48 to 5.64)	2020–2023	1.84 (−0.60 to 3.10)	2.77^b^ (2.26 to 3.22)
Peritoneal	2015–2017	−5.06 (−12.2 to 2.68)	2017–2023	−2.51^b^ (−3.38 to −1.21)	−3.16^b^ (−4.6 to −1.6)
**Vascular access for hemodialysis**					
Long-term catheter	2015–2023	4.26^b^ (3.10 to 5.44)			4.26^b^ (3.10 to 5.44)
Short-term catheter	2015–2023	7.61^b^ (4.90 to 10.37)			7.61^b^ (4.90 to 10.37)
Arteriovenous fistula	2015–2019	2.41^b^ (1.53 to 3.84)	2019–2023	−0.64 (−2.03 to 0.21)	0.86^b^ (0.47 to 1.26)
**Brazilian region**					
Midwest	2015–2019	3.39^b^ (1.64 to 7.45)	2019–2023	1.12 (−2.89 to 2.88)	2.25^b^ (1.36 to 3.14)
Northeast	2015–2023	3.64^b^ (2.81 to 4.48)			3.64^b^ (2.81 to 4.48)
North	2015–2023	5.01^b^ (3.49 to 6.53)			5.01^b^ (3.49 to 6.53)
Southeast	2015–2020	2.07^b^ (0.179 to 2.88)	2020–2023	0.98 (−0.04 to 1.55)	1.66^b^ (1.44 to 1.90)
South	2015–2021	2.82^b^ (2.24 to 5.20)	2021–2023	−0.06 (−2.60 to 2.14)	2.09^b^ (1.53 to 2.77)
Positive serology for HIV	2015–2023	7.77^b^ (4.93 to 10.78)			7.77^b^ (4.93 to 10.78
Positive serology for hepatitis B	2015–2023	2.24 (−1.90 to 6.52)			2.24 (−1.90 to 6.52)
Positive serology for hepatitis C	2015–2023	−3.27^b^ (−4.89 to −1.57)			−3.27^b^ (−4.89 to −1.57)
Recovery of renal function	2015–2023	5.96^b^ (3.36 to 8.64)			5.96^b^ (3.36 to 8.64)
Patients transferred between centers	2015–2020	0.13 (−10.15 to 4.53)	2020–2023	12.5^b^ (3.75 to 26.21)	4.63^b^ (1.88 to 6.88)
1-yr mortality	2015–2021	5.24^b^ (2.49 to 15.98)	2021–2023	−8.53 (−18.89 to 1.55)	1.59 (−0.94 to 4.81)

Annual percentage change and average annual percentage change estimates by joinpoint regression. AAPC, average annual percentage change; APC, annual percentage change; CI, confidence interval.

a Segments in joinpoint regression represent periods with a consistent rate of change between identified joinpoints, indicating shifts in the trend.

b Indicates that average annual percentage change or annual percentage change is significantly different from zero at the *α*=0.05 level.

**Figure 3:**
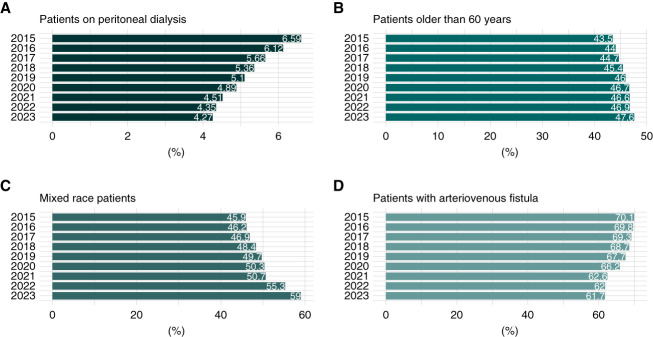
**Patients on KRT in the Brazilian Public Health System over the years (2015–2023).** (A) Percentage on peritoneal dialysis. (B) Percentage of patients aged 60 years and older. (C) Percentage of mixed race patients. (D) Percentage with arteriovenous fistula.

The use of peritoneal dialysis decreased from 6.6% to 4.3% over the years, AAPC −3.16% (95% CI, −4.6% to −1.6%) (Figure 3). There was an increase in patients using long-term catheters, AAPC 4.26% (95% CI, 3.10% to 5.44%), and short-term catheters, AAPC 7.61%. Most patients underwent KRT in the Southeast region, followed by the Northeast and South regions. The percentage of patients with positive HIV serology increased from 0.9% to 1.2%, AAPC 7.77% (95% CI, 4.93% to 10.78%). There was a reduction in the percentage of patients with positive hepatitis C serology from 3.4% to 1.9% over the years, AAPC −3.27% (95% CI, −4.89% to −1.57%) (Tables 1 and 2).

The median (IQR) reimbursement value of the maintenance dialysis procedure ranged from US$ 6793 (2681–7401) to US$ 6906 (3125–7403) dollars per patient per year (Table 1). The total expenditure for the entire dialysis population in the year 2023 was $835,794,206 US dollars.

**Figure 4:**
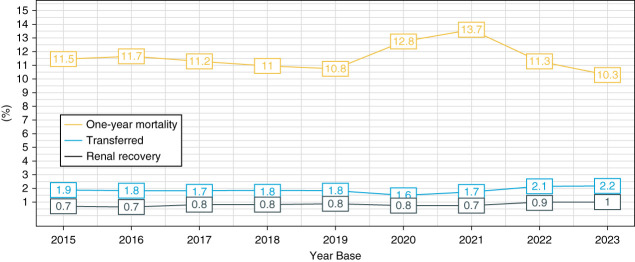
Patients on KRT in the Brazilian Public Health System: trends from 2015 to 2023, stratified by 1-year mortality, renal function recovery, and intercenter transfers.

**Figure 5:**
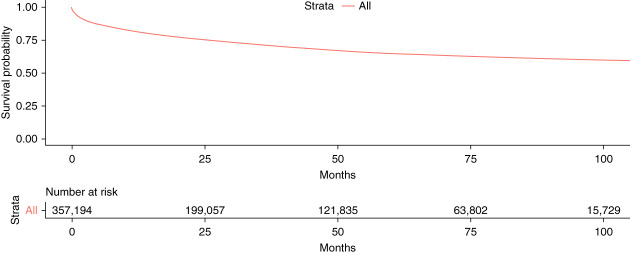
Survival analysis of incident KRT patients in the Brazilian Public Health System using the Kaplan–Meier method.

The crude 1-year mortality in prevalent patients ranged from 10% to 14%, remaining stable at around 10%–11% per year, except during the years of the COVID-19 pandemic when it increased to 13%–14% per year (Figure 4). For incident dialysis patients, the survival rates at 12, 24, 36, 60, and 96 months were 81%, 75%, 71%, 65%, and 60%, respectively (Figure 5).

The overall estimated annual prevalence rate of dialysis increased from 654 to 792 per million population over the years. The overall number of dialysis centers in the country also increased from 692 to 716, and the number of Brazilian cities with dialysis facilities rose from 407 to 449 (Table 3). Incident patients ranged from 26% to 28% of prevalent patients each year.

**Table 3:** Total number, Brazilian population, and prevalence rate of dialysis-related KRT in the Brazilian Public Health System over the years ranging from 2015 to 2023

Year	Prevalence of KRT, No.	Incident KRT^a^, No.	Brazilian Population^b^	Prevalence per 1,000,000^c^	Dialysis Centers (*n*)	Brazilian Cities (No.) with Dialysis Centers
2015	133,786	37,065	204,450,049^b^	654	692	407
2016	135,680	36,692	206,081,432^b^	658	687	407
2017	139,205	36,663	207,660,929^b^	670	693	412
2018	143,660	37,613	208,494,900^b^	689	702	419
2019	149,701	39,841	210,147,125^b^	712	713	425
2020	153,683	39,173	211,755,692^b^	725	714	425
2021	156,428	43,987	213,317,639^b^	733	723	430
2022	156,862	42,253	203,080,756^d^	772	724	442
2023	161,685	43,887	204,136,776^e^	792	716	449

Patients undergoing KRT; absolute numbers.

a New patients starting KRT during the year.

b Brazilian population estimate https://sidra.ibge.gov.br/tabela/6579#resultado.

c Prevalence rate of dialysis patients per million population.

d 2022 Brazilian population census.

e Population estimate derived from the 2022 census.

We observed a decline in the annual serum hemoglobin levels over time, from a median value of 10.82 g/dl (IQR, 9.42–11.92) to 10.53 g/dl (IQR, 9.11–11.57), AAPC −0.37% (95% CI, −0.54% to −0.17%). In addition, there was an improvement in spKt/V, which increased from a median of 1.14 (IQR, 1.00–1.50) to 1.34 (IQR, 1.09–1.59) per dialysis session over the study period, AAPC 1.23% (95% CI, 0.76% to 1.79%) (Tables 4 and 5).

**Table 4:** Biochemical examinations of patients undergoing KRT in the Brazilian Public Health System over the years ranging from 2015 to 2023

Characteristic	2015, *n*=133,786	2016, *n*=135,680	2017, *n*=139,205	2018, *n*=143,660	2019, *n*=149,701	2020, *n*=153,683	2021, *n*=156,428	2022, *n*=156,862	2023, *n*=161,685
Hemoglobin (g/dl)	10.82 (9.42–11.92)	10.64 (9.20–11.73)	10.50 (9.10–11.58)	10.33 (9.00–11.50)	10.49 (9.00–11.57)	10.50 (9.10–11.57)	10.29 (9.00–11.43)	10.37 (9.00–11.45)	10.53 (9.11–11.57)
Phosphorus (mg/dl)	5.00 (4.00–6.03)	4.92 (3.92–6.00)	4.91 (3.83–6.00)	5.00 (4.00–6.00)	4.92 (3.92–5.97)	5.00 (4.00–6.00)	4.88 (3.91–5.94)	4.82 (3.90–5.84)	4.86 (3.98–5.85)
PTH (pg/ml)	322 (160–594)	310 (156–563)	311 (157–558)	318 (162–563)	332 (170–598)	355 (187–639)	327 (165–614)	298 (139–586)	302 (143–590)
Single-pool Kt/V	1.14 (1.00–1.50)	1.15 (1.00–1.50)	1.17 (1.00–1.55)	1.20 (1.00–1.67)	1.25 (1.00–1.67)	1.25 (1.00–1.67)	1.26 (1.00–1.61)	1.35 (1.10–1.60)	1.34 (1.09–1.59)
Albumin (g/dl)	3.74 (3.00–4.00)	3.75 (3.00–4.00)	3.67 (3.00–4.00)	3.75 (3.00–4.00)	3.78 (3.00–4.00)	4.00 (3.20–4.00)	3.82 (3.08–4.00)	3.74 (3.26–4.00)	3.78 (3.36–4.02)

Kt/V (single-pool Kt/V−dialysis adequacy parameter). PTH, parathormone.

Values are median and percentiles 25% and 75%.

**Table 5:** Annual percentage changes in biochemical examinations of patients undergoing KRT in the Brazilian Public Health System over the years ranging from 2015 to 2023

Characteristic	Segment 1^a^	APC (95% CI)	Segment 2^a^	APC (95% CI)	AAPC (95% CI–Full Range)
Hemoglobin (g/dl)	2015–2018	−1.08^b^ (−2.01 to −0.50)	2018–2023	0.05 (−0.23 to 0.77)	−0.37^b^ (−0.54 to −0.17)
Phosphorus (mg/dl)	2015–2023	−0.12 (−0.77 to 0.61)			−0.12 (−0.77 to 0.61)
PTH (pg/ml)	2015–2023	1.60 (−2.23 to 5.52)			1.60 (−2.23 to 5.52)
Single-pool Kt/V	2015–2023	1.23^b^ (0.76 to 1.79)			1.23^b^ (0.76 to 1.79)
Albumin (g/dl)	2015–2023	0.86^b^ (0.31 to 1.54)			0.86^b^ (0.31 to 1.54)

Average annual percentage change or annual percentage change estimates by joinpoint regression. AAPC, average annual percentage change; APC, annual percentage change, CI, confidence interval; PTH, parathormone.

a Segments in joinpoint regression represent periods with a consistent rate of change between identified joinpoints, indicating shifts in the trend.

b Indicates that average annual percentage change or annual percentage change is significantly different from zero at the *α*=0.05 level.

**Figure 6:**
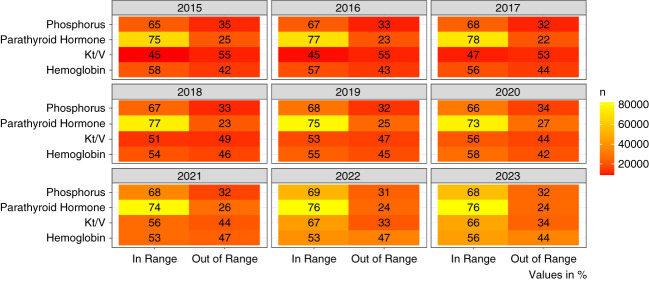
Monthly biochemical examinations bundle in KRT: trends in the Brazilian Public Health System from 2015 to 2023.

When evaluating the percentage of patients within the KDOQI ranges, we found a decrease in the proportion of patients in the target hemoglobin range, from 58% to 56% over the years. Conversely, there was an increase in the number of patients within the Kt/V ranges, from 45% to 66% (Figure 6 and Table 6).

**Table 6:** Value ranges of monthly examinations according to the Kidney Disease Outcomes Quality Initiative guidelines in patients undergoing KRT in the Brazilian Public Health System over the years ranging from 2015 to 2023

Characteristic	2015, *n*=133,786	2016, *n*=135,680	2017, *n*=139,205	2018, *n*=143,660	2019, *n*=149,701	2020, *n*=153,683	2021, *n*=156,428	2022, *n*=156,862	2023, *n*=161,685	*P* Value
**Hemoglobin**										<0.001
In range	20,886 (58%)	19,433 (57%)	19,860 (56%)	20,544 (54%)^a^	23,793 (55%)^a^	27,690 (58%)^a^	27,208 (53%)	39,717 (53%)	44,628 (56%)^a^	
Out of range	14,956 (42%)	14,791 (43%)	15,539 (44%)	17,428 (46%)	19,115 (45%)	20,391 (42%)	24,503 (47%)	35,204 (47%)	35,483 (44%)	
**Phosphorus**										<0.001
In range	22,763 (65%)	22,708 (67%)	24,454 (68%)	25,431 (67%)	29,821 (68%)	31,594 (66%)	36,320 (68%)^a^	53,432 (69%)	56,888 (68%)	
Out of range	12,241 (35%)	11,294 (33%)^a^	11,573 (32%)	12,253 (33%)	13,877 (32%)	16,454 (34%)	16,981 (32%)	24,556 (31%)	26,291 (32%)	
**PTH**										<0.001
In range	67,604 (75%)	70,611 (77%)^a^	73,038 (78%)	76,295 (77%)	80,122 (75%)	77,342 (73%)	80,585 (74%)^a^	81,883 (76%)	82,665 (76%)	
Out of range	22,052 (25%)	20,856 (23%)	21,078 (22%)	22,359 (23%)	26,467 (25%)	28,656 (27%)	27,858 (26%)	26,246 (24%)	26,738 (24%)	
**Kt/V**										<0.001
In range	9140 (45%)	9677 (45%)	11,221 (47%)^a^	13,927 (51%)^a^	18,320 (53%)^a^	21,662 (56%)	27,489 (56%)	44,102 (67%)^a^	47,510 (66%)^a^	
Out of range	11,351 (55%)	11,613 (55%)	12,559 (53%)	13,209 (49%)	16,180 (47%)	17,137 (44%)	21,494 (44%)	21,888 (33%)	25,011 (34%)	

Kt/V (single-pool Kt/V−dialysis adequacy parameter). KDOQI, Kidney Disease Outcomes Quality Initiative; PTH, parathormone.

Hemoglobin in range: hemoglobin level between 10 and 12 g/dl; phosphorus in range: serum phosphorus between 3.5 and 5.5 mg/dl; parathormone in range: serum level between 150 and 600 pg/ml; Kt/V in rage: levels higher than 1.2. The numeric variables were reported in median and percentiles (25% and 75%).

For categorical variables, we used the chi-squared test.

a Different from the previous year adjusted by Holm for multiple comparisons.

## Discussion

We evaluated an 8-year cohort of patients undergoing KRT by dialysis in Brazil funded by the Public Health System. We observed an increase in the absolute number and prevalence rate of patients from 2015 to 2023 in Brazil. During this period, we also noted an increase in the age at which the dialysis began and in the prevalence of mixed race patients, as well as a reduction in the proportion of those undergoing peritoneal dialysis and with arteriovenous fistula. In addition, we observed an increase in the range values of spKt/V over the years but a decrease in hemoglobin ranges. The 1-year crude mortality rate remained at 10%–11% except for the notable increase in the COVID-19 pandemic years.

The Brazilian Society of Nephrology Dialysis Survey provides compelling evidence and offers an estimate of the number of patients on dialysis in Brazil. It currently stands as the most reliable estimate of dialysis patients in the country despite several methodological limitations.^3^ The survey is based on an online questionnaire available to all Brazilian dialysis centers, with a response rate ranging from 28% to 38%.^3,12,13^ Therefore, the absolute number of dialysis patients in Brazil provided by this source is an approximation derived from the responding centers, which may be more susceptible to bias related to factors associated with participation. In addition, the survey is based on grouped patient data; the information is not individualized, and there is no patient follow-up.

The Brazilian Society Survey estimated the absolute number of patients on dialysis on July 1, 2022, to be 153,831. The estimated prevalence of patients reimbursed by private health insurance and the public health system was 19.7% and 80.3%, respectively.^3^ Accordingly, the number of patients on dialysis in the public health system was 123,526. We observed a total of 156,862 patients on dialysis during the year 2022 in the public health system. Assuming that 19.7% of patients received dialysis funded by private insurance, the total number of patients on dialysis during 2022 could reach 187,764. Our numbers provided a more accurate estimate because they were based on absolute figures obtained through a detailed analysis of mandatory report claims data from DATASUS containing individual data for each patient from the beginning of KRT and throughout its course. It is important to emphasize that the data from the Brazilian Dialysis Survey represents a point prevalence estimate on July 1, whereas the data in this report reflect the prevalence throughout the year, as obtained from DATASUS.

The overall number and prevalence rate of patients undergoing KRT per year increased during the studied period, except for 2022 during the COVID-19 pandemic when the number of patients remained steady. In addition, our prevalence rates from 2015 to 2021 underestimate the actual figures because they were based on inflated general population estimates by the IBGE during this period. In 2023, we observed a prevalence of KRT by dialysis of 792 per million population (pmp). By adding the patients with a functioning graft (approximately 60,000), the overall KRT prevalence in Brazil reaches 1047 pmp.

We observed an increase in the average age of dialysis initiation over the years, ranging from 57 to 59 years, with a higher percentage of patients older than 60 years, ranging from 43% to 48%. Brazil is facing an aging population, because the number of people 65 years and older has increased by 57%, from 14.1 million in 2010 to 22.2 million in 2022, now representing 11% of the total population.^15^ Data from the European Renal Association in 2020 showed a median age at dialysis onset of 65.6 years.^16^

We observed a reduction in the proportion of chronic peritoneal dialysis utilization in Brazil over the years, with a decrease from 6.6% in 2015 to 4.3% in 2023. Worldwide, peritoneal dialysis represents 11% of all dialysis procedures.^17^ Data from the United States Renal Data System showed that 8.1% of all dialysis patients in 2022 were on peritoneal dialysis.^18^ A significant limitation is the disparity in financial incentives for dialysis providers and nephrologists working in dialysis facilities, who receive lower fees per procedure for delivering peritoneal dialysis compared with hemodialysis. In addition, Brazilian nephrologists often lack the necessary training to effectively manage peritoneal dialysis programs.^19^

The official classification of race/skin color in Brazil is composed of five categories—White, Brown, Black, Yellow, and Indigenous.^20^ The distribution of ethnicity among Brazilian people according to the IBGE 2022 was as follows: mixed race (Brown/Black), 55.5%; White, 43.5%; and Yellow/Indigenous, 1%.^21^ We found a similar distribution in the KRT population in 2022 as follows: Brown/Black, 55%; White, 39%; and Yellow/Indigenous, 2.1%. In recent years, fewer patients have been classified as not informed, and many of them may have been correctly classified as mixed race, which may partly explain the significant differences observed in our study over time.

We observed a reduction in the percentage of patients with arteriovenous fistula over the years, mainly due to an increase in long-term dialysis catheter use. This finding aligns with the Brazilian census report, indicating that the central venous catheter prevalence increased from 15.4% to 23.9% between 2013 and 2021.^12^ Difficulties in accessing surgical procedures in public health care could be a possible explanation for the reduction in fistula use over the years.^22^

Anemia remains an unsolved problem for dialysis patients. We found that only 56% of patients in 2023 had achieved the values recommended in the KDOQI guidelines, with a median hemoglobin level of 10.5 g/dl. These results did not improve over the years. In Brazil, essential and high-cost medications used for patients with CKDs, such as erythropoietin and phosphorus binders, are provided free of charge by the public health system. However, some of these medications were unavailable in the state pharmacies,^23^ potentially contributing to not reaching the ranges recommended by the KDOQI for anemia. By contrast, the proportion of patients within the recommended ranges for phosphorus and parathyroid hormone was higher in 2023 (68% and 76%, respectively), denoting the greater expertise of nephrologists in treating bone disease and secondary hyperparathyroidism.

We observed an improvement in spKt/V in hemodialysis patients over the years. Only 45% of patients were in KDOQI ranges in 2015, compared with 66% in 2023. This is important because a Kt/V above the 1.2 threshold has been associated with improved survival in hemodialysis.^24^ The improvement could be attributed to better hemodialysis practices over time, including increased dialysis time and advancements in technologies.^25^

Mortality remains high among dialysis patients, with cardiovascular disease affecting more than two thirds of individuals receiving hemodialysis. It is the leading cause of morbidity, accounting for almost 50% of mortality.^14^ One-year mortality rates in prevalent patients were 6.6% in Japan, 15.6% in Europe, and 21.7% in the United States.^14^ Our study revealed a mortality rate ranging from 10% to 14% per year, with an excess in mortality observed during 2021–2022 associated with the COVID-19 pandemic. These figures may eventually be affected by the underreporting of mortality in the system as mortality data were not fully validated using other databases to crosscheck this information. For incident patients, the peridialysis study from northern Europe reported a first-year mortality of 19.3%,^26^ similar to our finding of 19%.

Total expenditure on kidney replacement procedures represented a significant portion of resources allocated to the public health system, corresponding to 4% of the annual budget of the Ministry of Health in 2014.^4^ The direct cost of kidney replacement procedures in 2023 was US$ 835,794,206. The Brazilian Government budget for health in 2023 was US$ 30.6 billion, representing 2.8% of the health expenditure in the same year. These expenses in this study do not include hospitalization costs or medications provided by the public health system, which would raise the costs to approximately 3.5%–4% of the government health budget. It is important to note the low reimbursement rate by the government for dialysis sessions in Brazil (US$ 6906 per patient per year in 2023), which is approximately half of that provided by private insurance companies, may have a negative impact on the overall care provided.

This study had some limitations. We were unable to retrieve data on the primary kidney disease or associated comorbidities: Most patient diagnoses were filled with the generic International Statistical Classification of Diseases and Related Health Problems, 10th Revision code N18.9, which indicates unspecified CKD. In addition, we could not retrieve data about patients on dialysis covered by private insurance because this information is not part of this public database. We also failed to retrieve monthly biochemical examinations for serum potassium because this information was not available in the dialysis reimbursement forms. The frequency of dialysis sessions was another missing information, but it was estimated from the procedure claims data. In the system, the maximum allowed number of sessions per month was 14 for adults (three sessions per week) and 18 for children (four sessions per week).

In conclusion, Brazil has a vast public health system that provides most of the complex procedures, including hemodialysis and peritoneal dialysis. This study analyzes the number and sociodemographic characteristics of patients undergoing dialysis funded by the public health system in Brazil over 8 years. We observed an increase in the age at dialysis initiation, a rise in the proportion of mixed-race individuals over the years, and a reduction in the use of peritoneal dialysis. Despite some improvements over the years, such as better hemodialysis adequacy as measured by spKt/V, correction of certain parameters, such as hemoglobin levels, remains challenging. The crude annual mortality rate over the 8 years consistently ranged between 10% and 11%, with a notable increase during the years of the COVID-19 pandemic.

## Data Availability

Anonymized data created for the study are or will be available in a persistent repository on publication. Recorded Data. Other. Harvard Dataverse. https://datasus.saude.gov.br.

## References

[1] Paim J Travassos C Almeida C Bahia L Macinko J. The Brazilian health system: history, advances, and challenges. Lancet. 2011;377(9779):1778–1797. doi:10.1016/S0140-6736(11)60054-8 21561655

[2] Barra ABL Silva APRda Canziani MEF Lugon JR Matos JPSde. Survival in hemodialysis in Brazil according to the source of payment for the treatment: public Healthcare System (SUS) versus private insurance. J Bras Nefrol. 2023;45(3):302–309. doi:10.1590/2175-8239-JBN-2022-0131en 36662571 PMC10697161

[3] Nerbass FB Lima HdN Moura-Neto JA Lugon JR Sesso R. Brazilian dialysis survey 2022. J Bras Nefrol. 2024;46(2):e20230062. doi:10.1590/2175-8239-JBN-2023-0062en 38078834 PMC11210532

[4] Sesso R Lugon JR. Global dialysis perspective: Brazil. Kidney360. 2020;1(3):216–219. doi:10.34067/KID.0000642019 35368628 PMC8809253

[5] DATASUS – Ministério da Saúde. Accessed July 21, 2022. https://datasus.saude.gov.br/

[6] Prestes IV de Moura L Duncan BB Schmidt MI. A national cohort of patients receiving publicly financed renal replacement therapy within the Brazilian Unified Health System. Lancet. 2013;381 [Suppl 2]:S119. doi:10.1016/S0140-6736(13)61373-2

[7] de Moura L Prestes IV Duncan BB Thome FS Schmidt MI. Dialysis for end stage renal disease financed through the Brazilian National Health System, 2000 to 2012. BMC Nephrol. 2014;15:111. doi:10.1186/1471-2369-15-111 25008169 PMC4099158

[8] Saldanha RdF Bastos RR Barcellos C. Microdatasus: pacote para download e pré-processamento de microdados do Departamento de Informática do SUS (DATASUS). Cad Saúde Pública. 2019;35(9):e00032419. doi:10.1590/0102-311x00032419 31531513

[9] National Kidney Foundation. KDOQI clinical practice guideline for hemodialysis adequacy: 2015 update. Am J Kidney Dis. 2015;66(5):884–930. doi:10.1053/j.ajkd.2015.07.015 26498416

[10] IBGE. 2022 Census. Accessed February 17, 2024. https://www.ibge.gov.br/en/statistics/social/labor/22836-2022-census-3.html

[11] Kim HJ Fay MP Feuer EJ Midthune DN. Permutation tests for joinpoint regression with applications to cancer rates. Stat Med. 2000;19(3):335–351. doi:10.1002/(sici)1097-0258(20000215)19:3<335::aid-sim336>3.0.co;2-z 10649300

[12] Nerbass FB Lima HdN Thomé FS Vieira Neto OM Sesso R Lugon JR. Brazilian dialysis survey 2021. J Bras Nefrol. 2023;45(2):192–198. doi:10.1590/2175-8239-JBN-2022-0083en 36345998 PMC10627134

[13] Sesso RC Lopes AA Thomé FS Lugon JR Santos DRD. Relatório do censo brasileiro de diálise de 2010. J Bras Nefrol. 2011;33(4):442–447. doi:10.1590/s0101-28002011000400009 22189808

[14] Bello AK Okpechi IG Osman MA, . Epidemiology of haemodialysis outcomes. Nat Rev Nephrol. 2022;18(6):378–395. doi:10.1038/s41581-022-00542-7 35194215 PMC8862002

[15] Kirby T. Brazil facing ageing population challenges. Lancet. 2023;402(10415):1821. doi:10.1016/S0140-6736(23)02561-8 37980907

[16] ERA. Annual Reports. Accessed March 2, 2024. https://www.era-online.org/research-education/era-registry/annual-reports/

[17] Cho Y Bello AK Levin A, . Peritoneal dialysis use and practice patterns: an international survey study. Am J Kidney Dis. 2021;77(3):315–325. doi:10.1053/j.ajkd.2020.05.032 32800844

[18] United States Renal Data System. Annual Data Report. Accessed December 6, 2023. https://adr.usrds.org/

[19] Abensur H. How to explain the low penetration of peritoneal dialysis in Brazil. J Bras Nefrol. 2014;36(3):269–270. doi:10.5935/0101-2800.20140039 25317607

[20] Travassos C Laguardia J Marques PM Mota JC Szwarcwald CL. Comparison between two race/skin color classifications in relation to health-related outcomes in Brazil. Int J Equity Health. 2011;10:35. doi:10.1186/1475-9276-10-35 21867522 PMC3189864

[21] IBGE. Ethno-Racial Characteristics of the Population. Accessed February 17, 2024. https://www.ibge.gov.br/en/statistics/social/population/17590-ethno-racial-characteristics-of-the-population.html

[22] Franco RP Chula DC de Moraes TP Campos RP. Health insurance provider and endovascular treatment availability are associated with different hemodialysis vascular access profiles: a Brazilian national survey. Front Nephrol. 2022;2:985449. doi:10.3389/fneph.2022.985449 37675012 PMC10479601

[23] Coube M Nikoloski Z Mrejen M Mossialos E. Inequalities in unmet need for health care services and medications in Brazil: a decomposition analysis. Lancet Reg Health Am. 2023;19:100426. doi:10.1016/j.lana.2022.100426 36950032 PMC10025415

[24] AlSahow A Muenz D Al-Ghonaim MA, . Kt/V: achievement, predictors and relationship to mortality in hemodialysis patients in the Gulf Cooperation Council countries: results from DOPPS (2012–18). Clin Kidney J. 2021;14(3):820–830. doi:10.1093/ckj/sfz195 33777365 PMC7986324

[25] Himmelfarb J Vanholder R Mehrotra R Tonelli M. The current and future landscape of dialysis. Nat Rev Nephrol. 2020;16(10):573–585. doi:10.1038/s41581-020-0315-4 32733095 PMC7391926

[26] Heaf J Heiro M Petersons A, . First-year mortality in incident dialysis patients: results of the Peridialysis study. BMC Nephrol. 2022;23(1):229. doi:10.1186/s12882-022-02852-1 35761193 PMC9235232

